# Detecting the Unseen: Understanding the Mechanisms and Working Principles of Earthquake Sensors

**DOI:** 10.3390/s23115335

**Published:** 2023-06-05

**Authors:** Bingwei Tian, Wenrui Liu, Haozhou Mo, Wang Li, Yuting Wang, Basanta Raj Adhikari

**Affiliations:** 1 Institute for Disaster Management and Reconstruction, Sichuan University-The Hong Kong Polytechnic University, Chengdu 610207, China; bwtian@scu.edu.cn (B.T.);; 2 Sichuan University-Pittsburgh Institute, Sichuan University, Chengdu 610065, China; liuwenrui@stu.scu.edu.cn (W.L.);; 3 Department of Civil Engineering, Pulchowk Campus, Tribuvan University, Lalitpur 44600, Nepal

**Keywords:** sensors, earthquake DRR, monitoring, early warning, search and rescue

## Abstract

The application of movement-detection sensors is crucial for understanding surface movement and tectonic activities. The development of modern sensors has been instrumental in earthquake monitoring, prediction, early warning, emergency commanding and communication, search and rescue, and life detection. There are numerous sensors currently being utilized in earthquake engineering and science. It is essential to review their mechanisms and working principles thoroughly. Hence, we have attempted to review the development and application of these sensors by classifying them based on the timeline of earthquakes, the physical or chemical mechanisms of sensors, and the location of sensor platforms. In this study, we analyzed available sensor platforms that have been widely used in recent years, with satellites and UAVs being among the most used. The findings of our study will be useful for future earthquake response and relief efforts, as well as research aimed at reducing earthquake disaster risks.

## 1. Introduction

An earthquake is one of the major natural hazards which destroys lives and properties; more than 522 major earthquakes have occurred in the 21st century, killing more than 430,000 people worldwide [1]. The earthquake phenomenon is sudden, and people cannot organize effective action in a short time because the seismic wave is transmitted to a certain point which spreads instantaneously, destroying houses and critical infrastructures [2]. In the early hours (4:17 a.m. local time) of 6 February, a 7.8-magnitude earthquake struck southeastern Türkiye and some parts of Syria. About nine hours later, a 7.5-magnitude earthquake along with more than 200 aftershocks took the lives of at least 59,000 people and injured more than 100,000 [3]. The United Nations Development Programme (UNDP) estimated that 1.5 million people in Türkiye lost their homes and nearly 500,000 houses must be rebuilt.

Seismic waves are divided into three types according to their mode of propagation: P-waves, S-waves, and surface waves. A P-wave travels in the Earth’s crust at a speed of 5.5~7 km/s. It makes the ground vibrate up and down and is less destructive. An S-wave propagates in the Earth’s crust at a speed of 3.2~4.0 km/s [4]. Surface waves (R and L waves) are mixed waves generated by P-waves and S-waves, which meet at the surface. Their large wavelengths and strong amplitudes are the main factors that cause strong damage to infrastructures. Despite numerous efforts to develop earthquake prediction technology, it is still in the initial stage and remains a challenging area of research [2]. Therefore, it is difficult to provide imminent prediction for most earthquakes to enable prompt rescue operations. The experiences during emergency management after a major earthquake have shown that the first 3 days or so after an earthquake are very critical for the smooth operation of rescue and relief. So, the first 72 h after an earthquake are often referred to as the “golden hours” for rescue. After three days, the chances of survival decrease significantly due to dehydration, lack of food, and exposure to the elements. For example, the 1999 Izmit earthquake in Türkiye was one of the deadliest earthquakes in recent history, with over 17,000 people killed and many more injured. During the first 72 h after the earthquake, search and rescue teams from around the world worked together to locate and save survivors [5]. Many of those rescued during this time had been trapped in small spaces without access to food, water, medical attention, and other essential resources. A similar situation happened in 2015 after an earthquake in Nepal, where thousands of people were buried under collapsed buildings and rubble [6]. In the first 72 h, rescue teams from around the world worked tirelessly to pull survivors from the wreckage [7]. Many of those saved during this time were found trapped under rubble in remote areas that were difficult to access.

In recent years, there has been significant progress in the development of new types of sensors that can be used in wide areas of earthquake monitoring, prediction, early warning, emergency commanding and communication, search and rescue, and life detection [8]. One particularly exciting development in this field is the use of sensor networks to create real-time earthquake monitoring systems. These systems can quickly alert populations in affected areas to incoming earthquakes, giving them precious moments to take protective actions.

However, there are gaps between sensors’ development and earthquake response practice, especially from monitoring to rescuing. For example, scientists have made great strides in their ability to provide more detailed information about the characteristics of earthquakes using equipment such as satellite sensors and drone-based sensors. Such kinds of information and technologies are practiced in different earthquake disaster scenarios. However, these kinds of practices are scattered, and comprehensive review literatures are scanty. Therefore, this article reviewed all available earthquake response practices to provide their strengths and limitations in disaster scenarios.

## 2. Classification Based on Application Time

The classification of sensors in earthquakes is largely based on their time period of application, such as seismic monitoring for long-term use, earthquake early warning, life detection during rescue, and network support that provides information support. This study classified the whole process of sensors according to their application times and principles (Table 1). The common life-detection sensors primarily use visible light and infrared detection equipment to record vibration signals to detect the position of trapped individuals. To obtain accurate life signals, most of these sensors are located on the ground to provide a close exploration perspective and avoid interference from background noise. The airborne radar electro-optical composite life-detection system, DN-UAV, realizes two life-detection methods, namely, landing detection and hover detection, through the UAV platform to obtain the position information and field environment of buried personnel in ruins. Earthquake early warning sensors typically require long-term detection to provide continuous earthquake early warning functions. The principle involves detecting sound, vibration signals, gravity, and other factors to detect the buried depth, morphology, and distribution rules of different elastic formation interfaces. A radon meter measures the concentration of radon in the air and dose rate to obtain seismic signals. Communication-support sensors transmit seismic information through radio signals, with their application scenarios mainly located in space. For instance, the transmission between ground communication systems or the use of artificial earth satellites as relay stations to forward radio waves takes place.

## 3. Classification Based on Function

A significant number of sensors are being used during emergency situations based on their function, flight time, and usefulness. The benefits and limitations of those sensors are reviewed and analyzed in the following sections and Table 2.

### 3.1. Life-Detection Sensors

Life-detection sensors are used to collect physiological, physical, and chemical information of trapped survivors to effectively identify their location immediately after a disaster [8]. Based on their principles and types of sensors used, life-detection technologies can be classified into acoustic life-detection techniques, optical detection techniques, radar life-detection techniques [9], and volatile organic compound (VOC) detection techniques [10,11].

Acoustic life-detection technology is used to locate trapped individuals by detecting cries for help, movements, tapping, and even small chest fluctuations during breathing [12]. Passive sensors that receive trapped people’s cries for help and knocking sounds have the advantage that rescue workers can hear these sounds and locate them if they are within the detectable range. However, the practical application of this technology requires sufficient experience from operators due to noisy sound at the earthquake site. Recent advancements in sensor technology have enabled the detection of the chest fluctuations of a trapped person during breathing by transmitting sound waves and analyzing reflected waves [13]. This approach has become more effective in recent years because acoustic signals can penetrate metal walls and detect stationary people through breathing movements alone without being disturbed by the remains of the victim [13,14].

Optical detection technology includes visible light detection and infrared detection technology. The optical detection technology involves using a small camera equipped with a light source connected by a flexible data transmission line to penetrate the aperture of a collapsed building and avoid moving it. One form of life-detection technology, also known as a Snake Eye (SE) life detector [15], can determine the position and living condition of trapped individuals while avoiding secondary collapse. Infrared detection technology uses the infrared characteristics of the human body to distinguish a human body from the surrounding environment. Currently, Unmanned Aerial Vehicles (UAVs) are becoming popular for collecting video information, audio information, infrared information, and other information at the scene of disaster areas synchronously. The collected data are further classified by operating software to analyze the images and audio in the video and determine the location and living state of personnel [16,17,18].

Radar life-detection technology is one of the most mature and widely studied life-detection technologies at present. It was used extensively in the 2008 Wenchuan earthquake in Sichuan, China, and in the 2023 Türkiye–Syria earthquake. The common radar life-detection system is divided into Continuous Wave (CW) and Ultra-Wide Band (UWB) radar life-detection systems.

A CW radar transmits a monophonic continuous wave signal to demodulate the phase change of the reflected wave and obtain the breathing and heart rate of the person [19]. This is because the phase change of the reflected waves is linearly proportional to the displacement of the chest caused by cardiopulmonary activity [20]. The UWB radar life-detection system emits pulsed microwave beams on the biological body. The beam reflects the echo pulse according to the circular sequence modulated by biological activity that extracts the parameters of the life signal through the digital signal processing system [21]. However, due to the radiation effect of electromagnetic waves on the human body and interference caused by the simultaneous use of multiple radar life detectors at the earthquake site, radar life-detection systems still have some limitations in their use.

Volatile organic compound (VOC) detection technology refers to identifying characteristic compounds in the exhaled air, blood, and urine of trapped individuals by determining the type and content of VOCs in the environment [22]. Breathing is considered a unique feature that can determine if a trapped individual is still alive [23] by detecting CO_2_ and O_2_ levels. Ion Mobility Spectrometry (IMS) and electronic sensors are common VOC life-detection instruments [24], where IMS separates these volatile organic compounds according to the difference in the drift velocity of the product ions in the inert buffer gas under the influence of an electric field [25]. The electronic sensor (also known as the electronic nose) uses an array of gas sensors to simulate animal olfactory organs to recognize odors [26]. VOC life-detection technology has some limitations, such as interference from dust and other particles at the rescue site, different VOCs of different groups of people (especially the VOCs of people trapped for a long time, with a lack of water and food), and the insufficient miniaturization of equipment [27].

### 3.2. Seismic Monitoring Sensors

Seismic monitoring sensors are essential for measuring abnormal activity and precursor signals of earthquakes [28]. They provide invaluable data on the position, depth, magnitude, onset time of shocks, and source mechanism of earthquakes, both before and after they occur.

Sensors play a crucial role in seismic monitoring and are used in various applications such as mobile gravity monitoring [29], electromagnetic wave signal detection [30], and cross-fault deformation measurement [31]. The first seismic network was established in California, USA, in 1929 using Wood–Anderson seismometers [32].

Modern seismic networks typically consist of broadband and strong-motion seismometers. Broadband seismometers have a wide recording capacity ranging from hundreds of seconds to hundreds of hertz. The Southern California Seismic Network (SCSN) is an exemplary seismic network that has grown from 7 seismometers in 1929 to over 600 seismometers in 2021 [33] Each station is now equipped with co-located, three-component broadband and strong-motion seismometers.

Mobile gravity monitoring is an effective technique for earthquake prediction and exploration, primarily for two reasons. First, changes in gravity directly reflect crustal deformation and variations in the focus medium during earthquake incubation [34]. Second, seismic activity is intricately linked to the spatial inhomogeneity and temporal discontinuity of gravity change.

Earthquake incubation and occurrence involve multiple stages, starting from stress accumulation to energy release. During the earthquake breeding process, stress builds up in the source, leading to the migration of material in the crust and changes in crustal density, which then affect the corresponding surface gravity.

One notable success story comes from China, where a forecast system was developed based on the principle of “A field, a network”. This system uses mobile gravity monitoring to predict earthquakes and has been successful in detecting abnormalities in gravity prior to several significant earthquakes [35]. Gravity monitoring and prediction are foundational for earthquake prevention and disaster reduction efforts. This involves the use of gravity sensors mounted on both ground-based instruments and satellites. By continuously monitoring changes in gravity, researchers can detect patterns and anomalies that may indicate the potential for an earthquake. This information can then be used to inform early warning systems and evacuation plans, potentially saving lives and minimizing damage.

Several countries have developed earthquake early warning systems using various techniques, including Japan, Mexico, China, and the USA [36]. Among them, the most advanced system is the Japanese REIS earthquake early warning system. REIS can accurately calculate the location and magnitude of an earthquake just 5 s after receiving the seismic wave signal. Additionally, it can estimate the source mechanism of an earthquake rupture within approximately 2 min [37]. It is important to note that Japan’s ability to develop such an advanced earthquake early warning system is due in large part to its dense seismic station network. In Japan, there is approximately one seismic station every 20 km, which provides the necessary data to accurately calculate an earthquake’s location and magnitude within seconds of receiving the seismic wave signal.

The Shake Alert earthquake early warning system in the USA is composed of six components, including the station observation system, data transmission system, data processing and alarm center, test and certification platform, information release system, and end-users. When an earthquake occurs, the system’s automatic rapid reporting system takes between 3 to 5 min to relay the relevant earthquake information to the appropriate authorities and end-users. This includes location, magnitude, and estimated shaking intensity based on the seismic waves detected by the network of monitoring stations [38].

The earthquake early warning system in Mexico City (SAS) is composed of four main components. (1) There is an earthquake detection system that employs 12 digital seismometers spaced 25 km apart within a 300 km coastal area of Guerrero. Each station is equipped with a microcomputer capable of determining the magnitude of an earthquake within 10 s. (2) There is a communication system with a very-high-frequency (VHF) central radio relay station and three ultra-high-frequency (UHF) radio relay stations that transmit seismic information to Mexico City within just 2 s. (3) The central control system, located in Mexico City approximately 320 km from the Guerrero Coast area, continuously receives seismic signals and automatically processes them to determine the magnitude and decide whether to issue an alarm. (4) The alarm issuance system issues warnings via commercial radio, and relevant departments are equipped with special receivers where trained personnel are responsible for receiving and coordinating disaster prevention activities [39].

A change in magnetic field can be taken as a precursor of an earthquake because the huge accumulations of crustal pressure may change the properties of the rock layer. This phenomenon affects its electrical conductivity, and the trapped gas accumulated in the formation will also produce an electric current to affect the geomagnetic activity [40]. Therefore, it is sometimes controversial to regard electromagnetic motion as an earthquake precursor. It is not clear yet, but the reasons might be as follows: (1) the signal is too weak and easily mixed with background noise to distinguish it, such as noise from nearby vehicles or small changes in solar activity that can be mistaken for geological disturbance signals; (2) accurate measurement equipment at a fixed position with enough statistical recordings are required to resolve reliable signals [41]. A number of researchers have used artificial noise signals for seismic wave velocity monitoring [42]. Artificial seismic noise is usually dominated by high-frequency body waves, providing a high spatial resolution. In addition, the location of artificial noise sources is often fixed (e.g., industrial operations) or moves along a fixed trajectory (e.g., trains and cars), which is easy to track and simulate the movement of noise sources [43].

Micro-electromechanical systems (MEMS) are devices or systems that combine microstructures, micro transducers, and micro-actuators with signal processing and control circuits [44]. Nowadays, these are commonly found in smartphones and laptops. These sensors are inexpensive and can be used to construct ultra-dense arrays. Additionally, MEMS sensors are known for their high accuracy, low power consumption, and robustness, which makes them ideal for use in harsh environments [45,46].

**Figure 1:**
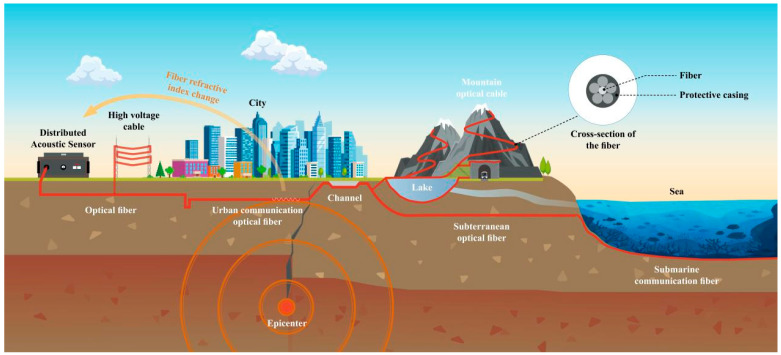
The operating wavelength profile of the different sensors.

Distributed Acoustic Sensing (DAS) is another effective technique to measure strain rate that consists of two parts, namely, a demodulator and sensing fiber optic cable (Figure 1). This fiber optic is deformed by the movement of the Earth’s crust, which causes the refractive index of the cable to change the phase of the back-scattered light [47]. The demodulator can detect seismic activity by analyzing the coherent Rayleigh scattered light phase information of the fiber [48]. Since 2017, DAS has emerged as a novel technology to obtain numerous seismic sensors at a relatively low cost. The concept of DAS was proposed in the 1990s, followed by being applied in various fields. However, its applicability in earthquake seismology has only recently been considered.

Post-earthquake monitoring is being carried out using audio signals to locate human targets in a hidden way [41,49], and it can be strengthened by using Wi-Fi and Long-Term Evolution (LTE) in future [50]. This sensor is small and monitors the environment in a narrow space by sensing different physical characteristics such as temperature, humidity, pressure, and vibration. The collected sensor data are first sent to the monitoring node based on ZigBee technology and then transmitted to the monitoring center together with the monitoring images. The results of physical experiments show that using these wireless sensors, the monitoring center can display the monitoring image of the monitoring area in real time and visualize the collected sensor data [29]. The ongoing research has been using intelligent monitoring algorithms (such as object recognition or intrusion detection) on monitoring nodes to achieve better monitoring performance [51]. Other advancements include the optimization of the mechanical design of the monitoring nodes (e.g., miniaturization or lightweight) and the positioning algorithms for the sensor nodes.

The co-seismic dislocation and optical data are the main parts of seismic monitoring via remote satellites [52], where GNSS and InSAR measure the co-seismic dislocation. Ground-based receivers using satellite signals from global navigation satellite systems (GNSS) such as the Global Positioning System (GPS) have served as primary sensors for over a decade to measure co-seismic ground deformation [53,54,55]. The combination of ground-based GPS and remote satellite information is very useful to improve earthquake deformation [56].

Synthetic Aperture Radar (SAR) is an imaging radar that uses a small antenna that moves at a constant speed along a trajectory of a long array and radiates coherent signals to process the echoes received at different locations coherently for a higher resolution [57]. Similarly, InSAR (Interferometric Synthetic Aperture Radar) is an advanced technique that combines synthetic aperture radar imaging technology with interferometry to measure the phase difference of two or more SAR images [58]. InSAR accurately measures the three-dimensional position and small changes in any points on the Earth’s surface and has been demonstrated to be a reliable tool for measurements [59]. 

The use of optical satellite data to detect various anomalies before a strong earthquake is the key to predict seismic activity because it can identify phenomena related to thermal radiation in the initial stage of an earthquake. Therefore, satellite observations are powerful tools for monitoring earthquake preparedness areas in near real time on a large scale [60].

### 3.3. Earthquake Early Warning

**Figure 2:**
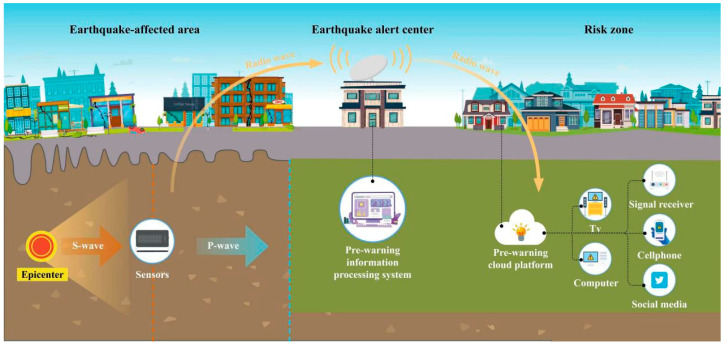
Principle of earthquake early warnings.

The main purpose of an earthquake early warning is to detect earthquakes in the early stages to estimate the seismic intensity of the expected area and warn users before the seismic waves spread to the ground [61]. The occurrence of an earthquake is sudden, and it is therefore not possible to predict accurately. However, a few seconds of warning can allow people to escape the building, find proper shelter, and move to a safer place inside the building [62]. An earthquake early warning system detects non-destructive seismic waves (P-wave) emitted at the beginning of an earthquake, while destructive seismic waves (S-wave) arrive at the surface several seconds later due to relatively slow propagation velocity (Figure 2).

A seismic sensor can sense the speed and acceleration signals caused by the ground movement and convert them into directly datable electrical signals [45]. Seismic sensors are widely used in in energy exploration, building quality detection, and geological detection, in addition to in earthquake detection [46,63]. The main observation data include temperature, pressure, and humidity [64].

Mechanical earthquake early warning technology is the most widely used earthquake early warning technology based on microelectromechanical technology (MEMS). This technology emphasizes ultra-precision machining with small size characteristics, making it well-suited for large-scale applications due to its low cost and low power consumption. [65]. The P-wave sensors are an essential component of earthquake early warning systems as they can detect the first seismic waves generated by an earthquake. Factors that affect the warning time provided by an early warning system include the distance from the epicenter of the earthquake and the speed of data transmission. In regions with dense sensor networks, accurate and timely data from multiple sensors can be captured to minimize the warning time. This can provide valuable time for people to take protective measures and reduce the potential damage caused by an earthquake [62]. The electrochemical earthquake early warning technology is based on the solution flows relative to the electrode, resulting in the corresponding change in the ion concentration gradient generating the electrical output. Therefore, the electrical signal output by the cell changes with the change in the input seismic motion. The transmission of seismic wave signals can cause electrochemical seismic sensors to perform well at low frequencies compared to others. The electrochemical seismic sensors have little mechanical noise, a small amount of thermal noise and low power consumption, thus having a high signal/noise ratio and a wide dynamic range [46,66].

The magnetic fluctuations at low frequency (0.01~10 hz), 10 to 100 Tesla (nT), occur hours or days before the earthquake. This fluctuation can be detected by a sensor composed of a composite Metglas-PZT-Metglas sensor of the magnetoelectric (ME) composite material [62]. The composite has two components: a ferromagnetic layer that responds to a magnetic field by generating mechanical strain, and a piezoelectric layer that converts mechanical strain into voltage. This sensor is very small, light, and cheap and works at room temperature [67].

The abnormal element detection technology, such as a radon detection sensor, is unique to earthquake early warning systems [66]. The content of radon varies with the temperature, pressure, and humidity. Radon has a half-life of 3.8 days, so it can be detected shortly after the basement fissure has formed [68,69]. A rise in radon concentration is a sign of the formation of new basement cracks. The cracks facilitate the flow of groundwater, allowing radon to escape [65].

### 3.4. Communication Support Sensors

An earthquake can destroy communication channels by collapsing mobile base stations and power lines. At the same time, traffic lines between the disaster area and the outside world can be blocked, meaning the victims in the disaster area cannot communicate with the outer world [70]. This creates a great challenge in search and rescue operations. Special network service avenues can play a critical role in the first 72 h after an earthquake, in which conventional communication services are disrupted. These network service categories of earthquake relief sensors can be classified into wireless emergency communications and wired emergency communications.

#### 3.4.1. Space Satellite Communications

The communication satellite plays an important role in earthquake rescue because of its large communication range and good communication effect. It can be quickly deployed and opened within a short period of time and has the working characteristics of mobility and flexibility, strong environmental adaptability, etc. Its communication network covers a large area, is in real time, and receives a lot of unexpected information, which can provide information and communication security services between all levels of command at the earthquake site and complete the earthquake emergency rescue work. Satellite communication is self-contained and has low power requirements, requiring only small generators or solar cells to be provided at the terminal for communication services [71].

#### 3.4.2. Ground-Based Electromagnetic Wave Communication

Ground-based wave communication uses terrestrial electromagnetic waves that provide services for earthquake relief. This includes shortwave, digital trucking, two-way radio systems, microwave communications, and radio frequency identification devices (RFIDs). The most obvious application is maintaining communication among the rescue team personnel on the ground to coordinate their efforts.

Shortwave

Shortwave waves have strong penetrating abilities and can pass through mountains, buildings, and other obstacles because they have waves with a frequency of 3 to 30 MHz [72]. They are commonly used not only for long-distance communication such as maritime, aviation, and overseas communications but also for emergency communications, such as earthquakes, floods, and other disaster events. This is instrumental during emergencies because of the characteristics of simple equipment and a simple point-to-point communication platform [72].

Digital clusters

Digital clusters usually consist of multiple nodes, each of which is a computer, which are connected to each other through a high-speed network. This communication can be dynamic networking and emergency calls with data transmission using fax and voice service functions with automatic monitoring and alarm functions, etc. Therefore, it has become an important part of emergency communication and command and dispatch systems [73]. The digital trucking system can meet the command and mobilization requirements of a rescue department in the process of disaster relief because this can relate to satellite positioning and other functions.

Two-way radio system

Two-way radio has both transmitting and receiving functions that can be used for long-distance communication such as maritime and aviation communication, because it can enable two-way communication. Users can send and receive communications via radio waves, which are mainly used for the use of walkie-talkies when internet-based systems fail [74]. It is very important to organize rescue teams and coordinate operation and communication support for rescuers in the event of a communication breakdown caused by an earthquake.

Microwave Communication System

A microwave communication system uses waves between about 1 mm and 1 m with shorter wavelengths and higher frequencies. Microwave radio waves are highly resistant to interference and can transmit a large amount of information in a limited frequency band. Microwave communication plays a vital role during earthquake emergencies because an earthquake can destroy wired transmission networks such as fiber optic communication networks [75]. Through communication rescue, vehicles, and other carriers can quickly reach the disaster area and provide communication services. Satellite communication is also a kind of microwave communication located in space to achieve microwave relay communication.

Radio Frequency Identification Technology (RFID)

This is a non-contact automatic identification technology that identifies information about an item using radio frequency signals without the need for direct contact. RFID systems include readers and tags, where a tag is a chip that is implanted or attached to an item and has the function of storing information, and the reader is a device that can read the tag information via radio frequency signals. It has benefits over bar codes in terms of non-optical proximity communication, information density, and bidirectional communication capability [76]. In the rescue process after an earthquake, rescuers need to find buried survivors as soon as possible. Using life detectors with RFID tags, buried survivors can be found quickly. During the rescue process after an earthquake, rescuers need to coordinate rescue operations to ensure rescue efficiency. The proper use of RFID can strengthen the rescue service, collecting information and providing effective information support [61].

## 4. Sensor Integration Platform

The integration and effective use of search and rescue technology require unique platforms, because these technologies do not solely rely on specific technology. Various platforms integrated with different technologies provide significant contributions during earthquake search and rescue operations. Some of the existing platforms are described below.

### 4.1. Earthquake Emergency Vehicle

Earthquake emergency vehicles are very important tools during search and rescue operations following earthquake disasters because they can supply vital instruments and logistics instruments. Different countries classify earthquake rescue equipment in different classes, such as rescue trucks, ambulances, firetrucks, mobile command centers, urban search and rescue vehicles, medical support units ets. For example, in Japan, the “Hyper Rescue” series of vehicles is used for earthquake emergency responses. These vehicles are equipped with specialized equipment such as medical supplies, cutting and excavation tools, and communication systems. They can also serve as mobile commanding and information collecting centers [77]. In the USA, the Federal Emergency Management Agency (FEMA) uses a variety of earthquake emergency vehicles, including large trucks and trailers that carry generators, communications equipment, and other supplies. The Los Angeles Fire Department operates specialized urban search and rescue (USAR) vehicles, which include cranes, bulldozers, and other heavy machinery [78]. In China, specialized earthquake rescue vehicles called “earthquake rescue vehicles” are used to transport rescue personnel and equipment to disaster areas. These vehicles are equipped with a variety of specialized tools and equipment such as stretchers, oxygen supplies, and search cameras. In Italy, the National Fire Corps operates a fleet of specialized vehicles for earthquake responses, including bulldozers, excavators, and cranes. These vehicles are used to clear rubble and debris and to search for survivors trapped under collapsed buildings. The China Earthquake Administration has categorized emergency equipment into eight categories, which include detection, search and rescue, medical, communication, assessment and information, logistics, and rescue vehicles. These categories can be further subdivided based on their specific application scenarios and functions. In order to meet local needs, certain emergency vehicles are being modified to serve specialized functions such as forward command vehicles and telemedicine consultation vehicles.

**Figure 3:**
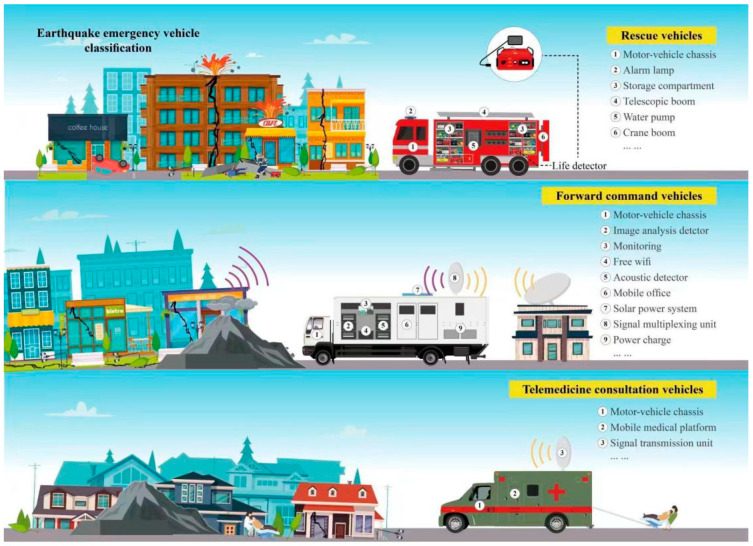
Three types of earthquake emergency vehicles.

Rescue vehicles equipped with life detectors, toxic and harmful gas detectors, communication equipment and basic medical equipment, including cardiopulmonary resuscitation machines and stretchers, have been developed to meet the needs of earthquake disaster site rescue (Figure 3). They can also supply power for other search and rescue equipment. Similarly, the forward command vehicle serves as an important channel for the collection and sharing of front-line information, providing an information channel for disaster assessment to have effective decision making and on-site resource scheduling. It mainly provides satellite communication, field voice communication, network communication, field network system, shortwave radio, and other communication functions. The telemedicine consultation vehicle is used as field medical and health equipment in the earthquake-stricken area where medical resources cannot reach the disaster area. This acts as an online platform and provides support through virtual consultation to the injured people in the hard-hit area.

### 4.2. Unmanned Vehicle

Unmanned vehicles are very useful in an area where an earthquake has damaged critical infrastructures or areas with toxic gas or polluted air which is unsafe and unhealthy [79]. These vehicles can reduce the threat of unknown environments to rescuers and emergency workers. Common unmanned devices include drones and mobile robots [19], but drones are the most used in disaster areas [79]. An unmanned aerial vehicle (UAV), a drone, can monitor a large area in a short period of time. Therefore, drones have become an increasingly popular tool for use in earthquake response and recovery efforts, including search and rescue operations, damage assessment, mapping, monitoring, infrastructure inspection, delivering aid, etc. [80]. They were widely used to provide long-term light in dark evenings and communication networks in search and rescue sites during the 2023 Türkiye MS 7.8 earthquake. A UAV also can create 3D maps of its surroundings using lasers, which are very useful for mountain environments.

Medical robots are very popular nowadays due to increasing accuracy. They can be divided into urban search and rescue robots, evacuation robots, and on-site diagnosis robots based on their functions. An urban search and rescue (USAR) robot, represented by the serpentine robot, conducts a preliminary exploration of the disaster site and identifies human survivors by examining the video (with audio) [81]. This instrument transmits the location of survivors to a centralized cloud server. It also monitors the relevant air quality in the selected area to determine whether it is safe for rescuers to enter the area [18]. An evacuation robot can be utilized to remove survivors from debris. An on-site diagnosis robot can judge the condition of the injuries of a survivor according to the skin condition of the buried person [82]. However, earthquake rescue robots are not popular due to their high cost, and they can only be brought to a disaster area by an emergency communication agency after the disaster. Therefore, it is difficult to use mobile robots dedicated to disaster reduction to carry out disaster reduction activities in the initial stage of a disaster.

### 4.3. Base Station

A base station receives and sends signals and forwards them to other terminal equipment. The composition of a mobile communication base station mainly includes a communication tower, antenna feeder system (antenna and feeder), communication room, main equipment, supporting facilities and equipment (grounding system, power supply system, lightning protection facilities, transmission equipment, transmission lines, air conditioning, alignment frame, lighting and monitoring facilities, and fire prevention facilities). It mainly consists of a rack, desktop, and self-supporting equipment. The main structural room is generally built with a reinforced concrete frame structure, brick, and color steel plate. The communication tower can be divided into the tower room (independent tower), outside tower, and roof tower.

A base station can be fixed and mobile. Fixed base stations are stationary and usually located in a specific location such as a roadside cellular base station. A mobile base station is very easy to deploy in an emergency situation due to its light weight and portable base stations in a vehicle-mounted base station.

The reliable operation of a communication network is important for the effective implementation of earthquake relief, and it is also a prerequisite for rescue teams to start a rescue smoothly. Different large earthquakes have destroyed communication channels around the globe in recent history. For example, the 2004 Indian Ocean earthquake and tsunami with magnitude of 9.1 caused damage to communication infrastructure in several countries, including India, Indonesia, and Thailand. The devastating 2010 Haiti earthquake with a magnitude of 7.0 caused extensive damage to the country’s communication infrastructure, including the destruction of the National Palace and many government buildings that housed important communication equipment. Communication was severely disrupted for weeks following the earthquake [83]. The Tohoku earthquake and tsunami (2011), which occurred off the coast of Japan, was one of the most powerful earthquakes ever recorded in Japan [7]. It caused widespread damage to communication infrastructure, including undersea cables and satellite systems. This led to major disruptions in internet and phone services. Similarly, according to a report by the Nepal Telecommunications Authority (NTA), a total of 1299 base transceiver stations (BTSs) were damaged or destroyed in the 2015 Gorkha earthquake [81]. In China, according to post-earthquake statistics, a total of 14,896 base stations were damaged in the 2008 Wenchuan earthquake; 724 base stations were disrupted in the 2013 Lushan earthquake; and 385 base stations were disrupted in the 2019 Changning earthquake. After destructive earthquakes, communication networks are affected and damaged to different degrees. For example, during the Wenchuan MS8.0 earthquake in 2008, communication base stations affected by the earthquake were located throughout the ≥VI intensity zone, and 29,064 base stations (including SCT base stations) were decommissioned and line damaged, resulting in communication interruptions.

Drones were deployed to survey damage and identify places where people were trapped in the Mw 7.1 Mexico City earthquake. The drones were equipped with thermal cameras to detect body heat under rubble and helped locate survivors in time to save their lives. Similarly, drones captured high-resolution images to assess the extent the damage caused by the 2015 Gorkha earthquake in Nepal [5]. These images also helped to create a high-resolution image for further geomorphic analysis. A 6.2 magnitude earthquake hit central Italy in 2016, and drones were quickly deployed to survey the area to identify victims under rubble. The drones were equipped with sensors to detect signs of life with the help of thermal imaging technology. After a series of earthquakes hit Puerto Rico in 2020, drones were used to survey damaged buildings and infrastructures [84]. The drones provided high-resolution images of damaged structures and buildings that were very useful in making important decisions for the local government [85]. In China, emergency rescuers used the mobile public network base station carried by the aerial emergency communication platform of the drone to provide continuous mobile communication signals. The multi-rotor drones provided instant calls, internet access, and other services 24 h a day during the Jiuzhaigou valley earthquake, China, on 8 August 2017. In addition, mobile base stations are very effective in situations where all kinds of communication channels are destroyed by an earthquake. For example, on 21 July 2021, an extraordinarily heavy rainstorm hit Zhengzhou city, China, destroying the electric communication equipment in many places and resulting in the interruption of all cell phone and network signals for the affected people. The emergency rescuers could not get information from the affected area. Therefore, the Ministry of Emergency Management urgently dispatched a winged dragon drone to fly long distances across the region.

### 4.4. Satellite

**Figure 4:**
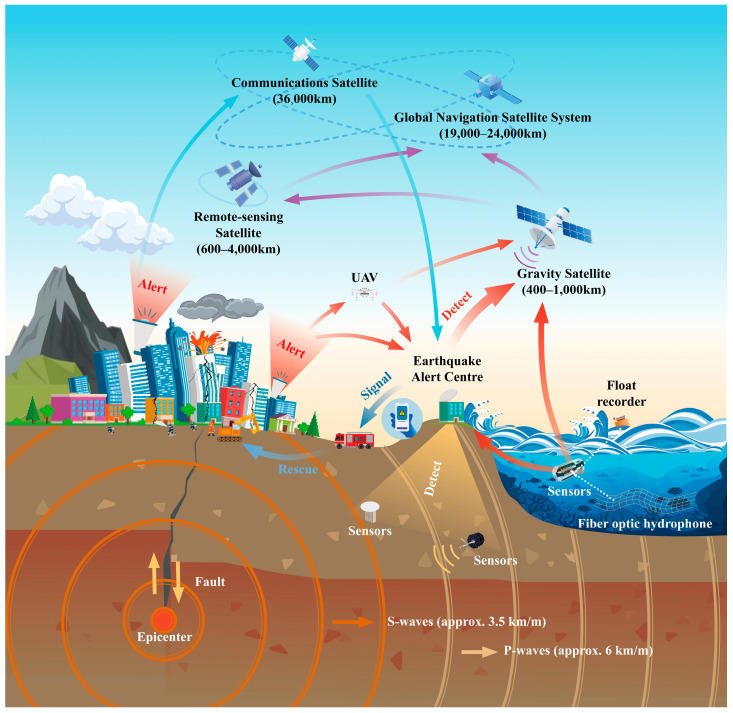
The mechanism of an earthquake and different kinds of sensors.

Satellites have many advantages during disastrous situations because they can provide a systematic and synergistic framework to facilitate scientific understanding of the Earth and thus facilitate disaster prediction and post-disaster support (Figure 4) [86]. Satellite data can help analyze the situation in a disaster area and provide rescuers with an important basis for decision making. They can further support earthquake rescue operations in the areas of detection, early warning, rescue navigation, communication, and the prediction of secondary hazards [87,88]. For example, the satellite imagery shared by the NASA Earth observatory showed the damage of Turkish cities after the destructive earthquakes occurred on 6 February 2023.

Navigation and monitoring satellites can perform detection and early warning functions before earthquakes. The recent advancement [52] of real-time high-rate GPS means this technology can directly estimate permanent displacement on the Earth. This information can be combined with ground seismic sensors to make a more accurate early warning of earthquakes [89]. Infrared satellites can predict earthquakes by detecting anomalous ground warming prior to an earthquake [90]. The satellite information can be used by different agencies on different levels. For example, satellites enable governments and rescuers to know about earthquakes in advance, send rescue teams, and evacuate residents [87,91,92]. For the public, satellites also allow people to know about earthquakes in advance so that they can take protective measures nearby.

Satellites provide historical and real-time images of the disaster area before and after the earthquake. Satellites also provide a unique synergistic view of the spatial scale and variable time of the disaster area. Earth-orbiting satellites complement traditional in situ measurements and ground-based sensor networks such as those for seismology, volcanology, geomorphology, and hydrology [93]. Satellite communication has the advantages of a large communication range, good communication effect, and not being influenced by terrestrial disasters, which plays an important role in earthquake rescue [93]. Moreover, satellites can provide the spatio-temporal information of a disaster area to understand the possibility of subsequent disasters [94]. This information can be used to avoid casualties from subsequent disasters and economic losses [95].

This review shows that there are benefits and limitations to earthquake sensors for decision making based on their model and applications (Table 3). Base stations, UAVs, and satellite sensors have different capacities and limitations because of their model design. For example, base station sensors have a very high cost compared to UAV sensors but they provide very good communication systems for long-term data for early warning systems. Meanwhile, UAV sensors are very good at detecting objects in harsh situations, but they have lots of technical challenges. Satellite sensors are very useful for large-scale disaster scenarios, but they are very expensive.

## 5. Conclusions

The application of earthquake sensors is very important for timely search and rescue operations in disaster scenarios. Earthquake sensors have been evolving in recent years and have been applied in different time and space scenarios. The development of frontier technologies such as the Internet of Things and Artificial Intelligence has provided unique opportunities in emergency situations. The current development status of seismic monitoring and rescue sensors is manifested in different aspects. The constant innovation in sensors technologies such as MEMS, DAS, UAV, satellite, and nanotechnology can enable to more effective detection and recording of seismic activity. Moreover, the use of big data and AI could help to achieve real-time data to share earthquake locations and destruction behavior with the public. This will enhance the capability of earthquake responders to provide early warning and rescue operations. Moreover, the new development of sensor networks could establish a stable communication network to achieve information transmission and real-time responses. The development of earthquake-related sensors is an ongoing process of innovation and expansion, with the continuous strengthening of scientific and technological progress, providing more efficient, intelligent, and comprehensive protection for earthquake rescue and relief. However, there is a strong need to strengthen the capabilities of earthquake sensors for timely prediction for effective disaster management. The integration of new innovative technology in earthquake prediction should be in place to provide comprehensive information sharing for effective disaster management in the future.

## Figures and Tables

**Table 1:** Modern sensors for earthquake monitoring, early warning, search, and rescue.

Example	Type	Application Time	Theory	Height of Application
Snake Eye Life detector	Life detector	0–72 h	Intercom probe, video probe, and infrared thermal imaging probe	Ground
LEADER Hasty	Life detector	0–72 h	It incorporates vibration signal listening, video searches, and calls to trapped people	Ground
Airborne radar-photoelectric composite life-detection system	Life detector	0–72 h	Two-dimensional radar and visible/infrared cameras	Low altitude
Native safety radar vibration compound life detector YSF40	Life detector	0–72 h	Intrinsically safe two-dimensional radar is combined with intrinsically safe wireless micro vibration sensor	Ground
Prototype of portable locator employing chemical analysis	Life detector	0–72 h	Infrared (IR) camera and different gas sensors	Ground
DZQ 12-3A cascade engineering seismograph	Seismic prospecting	Long period	Using hammer strike, electric spark, or explosion as the source, it is aroused by observing and recording the travel time of seismic wave and detecting the buried depth, shape, and distribution law of different elastic stratum interface, so as to solve the problem of engineering and resource geophysical exploration and explore depths from several meters to hundreds of meters	Underground: several meters to several kilometers
P-alert	Earthquake early warning	Long period	Earthquake P-wave sensor, in addition to the traditional S-wave detection function, but also embedded in the earthquake rapid report technology, can detect P waves and determine a catastrophic earthquake within 3 s.	Underground
Alpha GUARD	Earthquake early warning	Long period	Seismic precursor analysis using atmospheric radon anomaly.	Ground
CG-5 Gravimeter	Seismic prospecting	Long period	Studies the physical phenomena of gravity changes on the Earth’s surface and in the space around it.	Ground
Distributed acoustic sensing, DAS	Earthquake early warning	Long period	The acoustic sensor detects the external signal in the optical fiber. By extracting and demodulating the interference signal of sound vibration at different times, the quantitative measurement of external physical quantity can be realized.	Underground
Radio-Frequency Identification (RFID)	Communication support	0–72 h for saving lives and after 72 h for other usages	RFID is a generic term for technologies that use radio waves to automatically identify people or objects.	Ground
Two-way radio system	Communication support	Provide the possibility of communication after an earthquake	A two-way radio is a radio that can both transmit and receive radio waves (a transceiver), unlike a broadcast receiver which only receives content. It is an audio (sound) transceiver, a transmitter and receiver in one unit, used for bidirectional person-to-person voice communication with other users with similar radios.	Above/below ground
Communications Satellite	Communication support	Long period	Satellite communication is the communication between radio communication stations on Earth (including the ground and the lower atmosphere) using satellites as a medium.	Upper air
UAV	Communication support	Provides communication after an earthquake	It is equipped with photoelectric detection pod, synthetic aperture radar, aerial CCD camera, emergency communication support pod, emergency delivery pod and other equipment to provide communication support and obtain front-line information.	Up to 9000 m

**Table 2:** The benefits and limitations for the monitoring and detection of the different kinds of sensors.

Main Categories	Sub Categories	Benefits	Constraints
Life Detection	Acoustic	Non-invasive and can penetrate metal walls. Real-time, portable, and wide range.	Limited applicability High false positive rate Limited specificity
	Optical	Non-invasive, real-time, and portable. Provides highly accurate measurements of vital signs to avoid moving a collapsed building.	Limited scope Environmental sensitivity
	Radar	Non-invasive, real-time, and robust. Radar can sense through walls and other barriers that might block an optical signal.	Limited scope Privacy concerns
	Volatile organic compounds (VOCs)	Non-invasive and sensitive. VOC detection can be used in a wide range of environments, including air, water, soil, and rocks.	False positives Lack of specificity Sampling difficulties
Seismic monitoring	Gravity	Non-invasive and cost-effective. Gravity seismic monitoring can provide high-resolution data on subsurface geology and fluid movements.	Limited sensitivity Limited depth range Limited temporal resolution Technical challenges
	Micro-electromechanical systems (MEMS)	Cost-effective, compact size, and low power consumption. MEMS sensors are small and lightweight, which makes them easy to install and transport in wide areas. MEMS sensors offer high-resolution data that can capture small movements in the ground.	Limited frequency range Limited lifespan Sensitivity to environmental factors
	Distributed Acoustic Sensing (DAS)	Continuous monitoring and low power consumption. DAS offers high-resolution data that can capture small movements in the ground.	High cost Limited frequency range Calibration requirements Sensitivity to environmental factors
	Wi-Fi	Cost-effective, easy installation, and continuous monitoring. Wi-Fi sensors require minimal maintenance, as they are not affected by environmental factors such as temperature changes and humidity.	Limited range Limited resolution Power consumption Vulnerability to interference by other wireless devices
	Long-Term Evolution (LTE)	Cost-effective, remote access, and real-time data transmission. LTE networks cover large areas, which makes it possible to collect seismic data widely.	Limited data quality Dependence on cellular networks Lack of historical data
	Synthetic Aperture Radar (SAR)	Wide range for tracking the movement of ground. High-resolution images. Generates interferograms and 3D models of the Earth’s surface.	Affected by atmospheric conditions Data processing complex and time-consuming
Earthquake early warning	Mechanical	Rapid notification and accuracy. Mechanical sensors to detect the initial shock-waves of an earthquake and accurately determine its location, magnitude, and expected ground shaking intensity.	Limited coverage False alarms Time delay High cost
	Electrochemical	Quick, portable, and low cost. Detects multiple gases simultaneously, which can provide additional information with a high signal/noise ratio about the type and severity of an earthquake.	Limited range Limited detection capability False alarms
	Radon	Low-cost and long-term monitoring. Sensitive to precursory events. Radon sensors do not require any external power source or maintenance once installed.	Limited range Slow response time False alarms Limited applicability to certain geological settings
Communication support	Shortwave radio	Wide coverage, low cost, and reliable. Shortwave radio does not rely on any centralized infrastructure or service provider, which means it can operate independently, even when other systems are down.	Limited bandwidth Vulnerable to interference Reliance on specialized equipment Communication delay
	Digital trucking	Real-time updates and increased efficiency. Digital trucking can improve coordination among emergency responders by providing a centralized platform for tracking the location and movement of vehicles.	Dependence on technology Limited coverage High cost
	Two-way radio systems	Immediate communication and long range. Two-way radios are more reliable than cellular phones during earthquakes, as they do not rely on cellular networks that may become congested or fail during emergencies.	Limited coverage area Interference by other electronic devices Complex setup
	Microwave communications	High-speed and long-distance coverage. Microwaves are less prone to interference from natural obstacles such as mountains or valleys, making them more reliable for transmitting messages during an earthquake.	Vulnerability to weather conditions such as heavy rain Susceptibility to interference
	Radio frequency identification devices (RFID)	Real-time monitoring and scalability. RFID allows for automatic identification and tracking of objects, which can save time and effort during emergency response operations.	Limited range Security concerns High cost

**Table 3:** The benefits and limitations of earthquake sensor integration platforms for decision making.

Main Categories	Sub Categories	Benefits	Constraints
Earthquake Emergency Vehicles	Rescue	Quick response time and mobility.	High cost and limited use
	Commanding	Emergency communication systems.	Maintenance challenges
	Telemedicine	Equipped with specialized tools.	Safety concerns
Base Stations	Fixed	Long-term and immediate monitoring data for early warnings.	Damaged after destructive earthquakes and false alarms
	Mobile	Light weight and easy and quick to deploy monitoring and analysis.	Limited coverage, technical challenges, and cost
Unmanned Vehicles	Mobile robot: urban search and rescue snake robots, evacuation robots, on-site diagnosis robots, etc.	Decreases human risk. Conducts a preliminary exploration of the disaster site and identifies human survivors. Takes out the survivors from the debris. Monitors the relevant air quality, improves situational awareness, etc.	Limited coverage Technical challenges High cost
	Unmanned Aerial Vehicle (UAV)	Easy to transport for rapid survey and enhances situational awareness. Improves efficiency and can cover large area in short period of time. Provides long-term light in dark evenings. Creates 3D maps for monitoring.	Limited flight time Weather sensitivity Privacy concerns Technical challenges High cost
Satellites	Remote Sensing Satellite	Very wide area of coverage. Rapid response and provides historical and real-time images of disaster areas. High-resolution imaging. Long-term monitoring.	High cost Technical challenges Weather sensitivity except SAR Limited temporal resolution, depending on revisit period
	Communication Satellite	Real-time communication between disaster response teams. Large area coverage. Reliable communication.	High cost Technical challenges Limited bandwidth Weather sensitivity
	Global Navigation Satellite System	Real-time position for rescue and recovery. Large area coverage and cost-effective for earthquake monitoring.	High cost Technical challenges Weather sensitivity Limited vertical resolution

## Data Availability

All data generated or analyzed during this study are included in this published article.
